# Macro-financial models of Canadian dollar interest rate swap yields

**DOI:** 10.1371/journal.pone.0320132

**Published:** 2025-03-25

**Authors:** Tanweer Akram, Khawaja Mamun

**Affiliations:** 1 Independent Scholar,; 2 Longwood University; Rey Juan Carlos University: Universidad Rey Juan Carlos, SPAIN

## Abstract

This paper analyzes the dynamics of Canadian dollar–denominated (CAD) interest rate swap yields. It applies autoregressive distributive lag (ARDL) models, using monthly time series data, to estimate the effects of the current short-term interest rate on interest rate swap yields after controlling for relevant macro-financial variables. It shows that the current short-term interest rate is a crucial driver of the CAD swap yields of different maturity tenors. Previous empirical research testing the Keynesian hypothesis, which maintains that the current short-term interest rate has a decisive influence on the long-term interest rate, has discerned similar patterns for interest rate swaps denominated in other currencies. Thus, the findings of this paper lend additional support to the Keynesian hypothesis by showing that the same pattern holds for CAD interest rate swap yields.

## Introduction

Interest rate swaps are important fixed-income instruments not only for banks but also for other financial institutions and corporations. An interest rate swap enables the exchange of two cash flow streams based on certain interest rates. Usually, two parties exchange a stream of fixed interest rate payments and a stream of floating interest rate payments between each other; the payments are often in the same currency and based on a notional principal. Interest rate swaps are used both for hedging interest rate risks as well as for speculating on future interest rates. Previous research [1–8] has shown a connection between the short-term interest rate and the long-term swap yield in various currencies. An accessible primer on interest rate swaps is [9].

**Fig 1:**
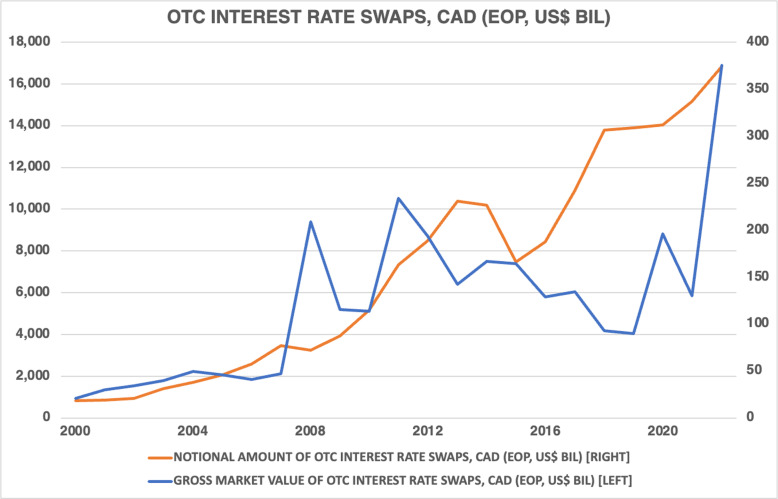
The evolution of Canadian dollar–denominated over-the-counter interest rate swaps, 2000–22.

Fig 1 shows, using data from [10], the evolution of outstanding over-the-counter Canadian dollar–denominated (CAD) interest rate swaps, giving both their nominal amount and gross market value. As of 2022, the notional value of CAD-denominated swaps amounted to $16.8 trillion (in US dollars [USD]) and the gross market value was $375 billion. The notional value of CAD interest rate swaps rose steadily through the period under study, except for a brief decline in 2014–15. The gross market value has since fluctuated, reaching a peak in 2022. The data displayed in fig 1 implies that CAD swaps are important financial instruments in CAD-denominated financial markets. Hence, econometrically modeling their dynamics could provide valuable insights for portfolio managers, corporate leaders, and policymakers.

In recent years there has been an advancement of empirical research that models interest rate swap yields from a Keynesian vantage point [1–8]. This research ties the long-term swap yield to the current short-term interest rate, after controlling for relevant macro-financial variables. Keynes [11–12] hypothesized on the connection between gilt-edged long-term government bond yields and the current short-term interest rate. Recent empirical research has shown that the connection appears to also hold for long-term yields on interest rate swaps denominated in several hard currencies, such as the USD, euro (EUR), British pound sterling (GBP), and Japanese yen (JPY).

This paper is part of an umbrella research project to model swap yields from a Keynesian perspective, as undertaken by [1–8]. It explores whether the Keynesian conjecture relating the long-term interest rate to the current short-term interest rate holds for CAD interest rate swap yields. Previously, [13] showed that the Keynesian conjecture holds for CAD government bond yields, but [13] did not explore the case for CAD interest rate swap yields. Given this critical lacuna, it is pertinent to delve into the relationship between CAD swap yields and the current short-term interest rate because it may provide insights into the monetary transmission mechanism in Canada and the effects of the Bank of Canada’s (BOC) monetary policy decisions on CAD-denominated financial markets.

The empirical modeling of swap yields from a Keynesian perspective constitutes a marked shift from the earlier literature, which was primarily focused on the credit, liquidity, and market technical conditions that affect swap yields rather than macroeconomic and financial market conditions to explain their behavior dynamics. This strand of the literature—exemplified by [14–20]—is not without some useful insights, but it failed to relate the effects of monetary policy, fiscal policy, effective demand, and national and global financial markets on swap yields. In contradistinction, this paper places the dynamics of CAD swap yields in their proper macroeconomic financial context by modeling them as a function of the current short-term interest rate and a host of other relevant macroeconomic and financial variables.

The paper proceeds as follows. First it provides the macroeconomic milieu to the evolution of CAD swap yields during the study period. Second, it describes the data and undertakes unit root and stationarity tests, which are available in the appendix, but not given here due to space constraints. Third, it presents the estimated econometric models and deliberates on their findings. It concludes with a summary and reflection on the policy implications of the findings.

## Macroeconomic milieu

Prior to undertaking the econometric modeling of CAD swap yields, it is worthwhile to have an overview of the swap yields’ macroeconomic milieu and its evolution during the study period.

**Fig 2:**
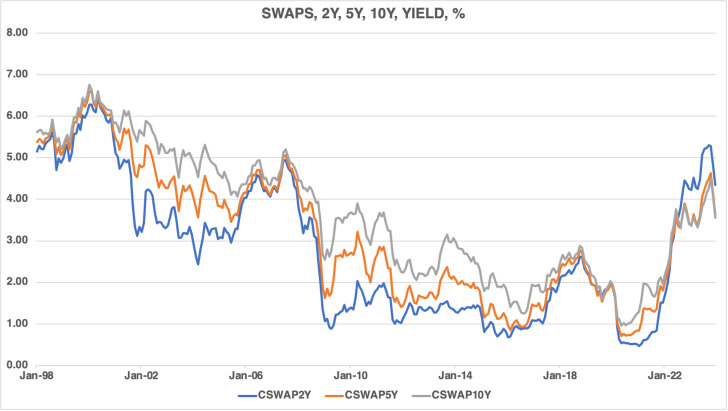
Canadian dollar interest rate swap yields, 1998M01–2023M12.

Fig 2 displays the evolution of CAD swap yields of 2-year, 5-year, and 10-year maturity tenors. The swap yields ranged between 5.0 percent to 5.6 percent at the beginning of the period, peaking in January 2000 and gradually declining until June 2005; this trend reserved and continued to rise until August 2007, followed by a steep decline until mid-2009. Swap yields stayed range-bound from mid-2009 until late 2018, but then declined in the subsequent months. With the onset of the global pandemic in early 2020, swap yields experienced a steep decline. In September 2022, swap yields began to increase as the BOC tightened its monetary policy.

**Fig 3:**
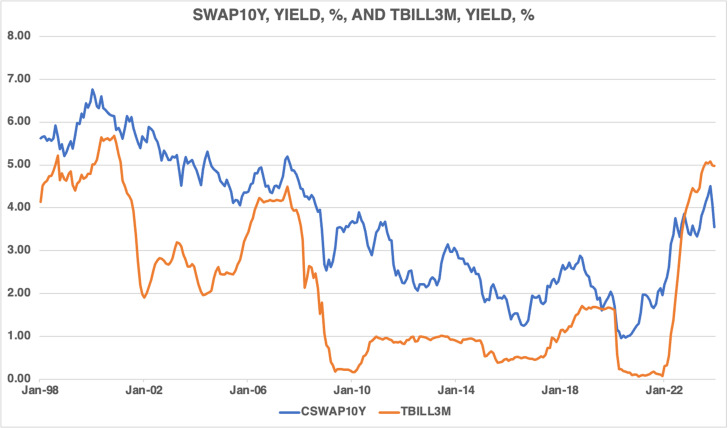
Canadian swap yields and the short-term interest rate on treasury bills, 1998M01–2023M12.

Fig 3 exhibits the evolution of the 10-year swap yield and the 3-month Treasury bill rate. It shows that the 3-month Treasury bill rate was lower than the 10-year swap yield. The 10-year swap yield tends to move together with the 3-month Treasury bill rate most of the time, but not always. Moreover, the swap yield appears to be more volatile than the Treasury bill rate during the period covered in this paper.

**Fig 4:**
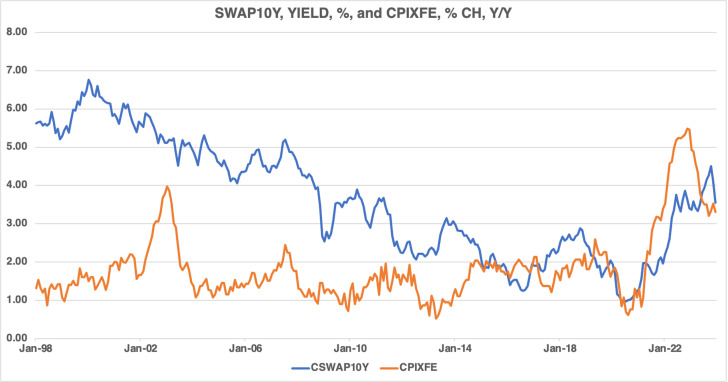
Canadian swap yields and core inflation, 1998M01–2023M12.

Fig 4 presents the evolution of the 10-year swap yield and core inflation. The correlation between the 10-year swap yield and core inflation is positive but weak. However, as core inflation rose following the global pandemic, the 10-year swap yield rose in tandem.

**Fig 5:**
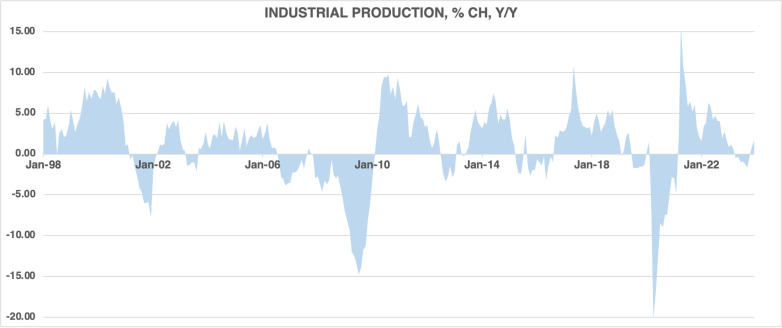
The growth of industrial production, 1998M01–2023M12.

Fig 5 depicts the growth of industrial production in Canada. Typically, industrial production grows; however, in recessionary periods, industrial production experiences slowdowns and outright declines, as shown in the recessions of 2001, 2008, and 2020.

**Fig 6:**
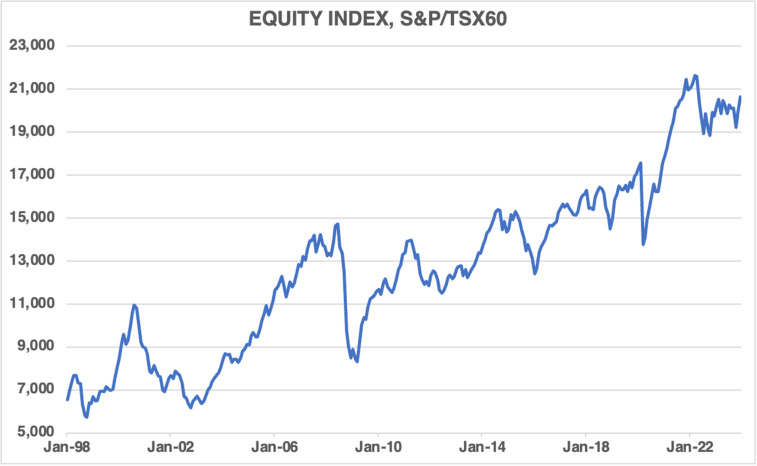
The S&P/TSX 60 equity index, 1998M01–2023M12.

Fig 6 charts the S&P/TSX 60 equity index. It shows that the index rose over the covered period, albeit with some volatility and periods of decline, particularly during recessions.

**Fig 7:**
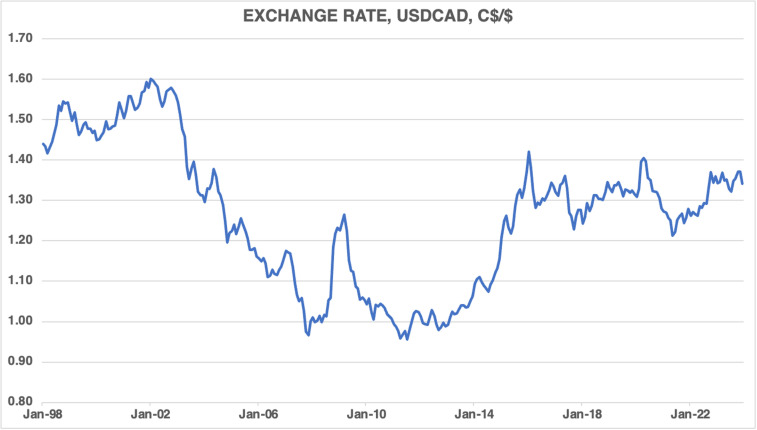
Exchange rate, CAD per USD, 1998M01–2023M12.

Fig 7 displays the CAD’s exchange rate against the USD. It shows that the CAD had depreciated against the USD to around $1.60 in 2002, followed by an appreciation until it reached parity in mid-2007. It appreciated in late 2007, followed by a depreciation to around $1.25 in late 2008. However, the CAD reached parity once again in early 2010 and remained stable against the USD until mid-2013. Subsequently the CAD depreciated until early 2016. Since then, the CAD has been stable—declining moderately in mid-2020 but stabilizing by mid-2021—and remained range bound between $1.20–1.30 against the USD.

### Data

This paper uses time series macroeconomic and financial data to model interest rate swap yields in Canada. In particular, the paper utilizes monthly data on various maturities of interest rate swap yields (SWAP), short-term interest rates (STIR), core inflation (INFL), economic activity (ACTIVITY), equity indexes (EQUITY), exchange rates (FX), and the size of the central bank’s balance sheet (BS). The data period covers from January 1998 through December 2023 and consists of 312 observations.

Interest rate swaps involves the exchange of interest rates between two parties. An interest rate swap is an agreement between two parties to exchange one stream of interest payments for another, over a predetermined period of time. The counterparties agree to exchange payments based on a defined principal amount, for a fixed period. However, in an interest rate swap, the principal amount is not actually exchanged between the counterparties; it is merely regarded as the notional principal of the swap. A floating to fixed rate swap allows an issuer with a variable rate debt to hedge the interest rate exposure by receiving a flexible rate in exchange for paying a fixed rate, whereas a fixed to floating rate swap allows the issuer with a fixed rate debt to take advantage of variable interest rates. The various terms of the CAD-denominated interest rate swaps are those of 2-year, 5-year, and 10-year maturities.

The short-term interest rates are based on 3-month and 6-month Canadian Treasury bills, which tend to closely follow the policy rates set by the BOC. Canadian Treasury bills are debt securities issued by Canadian federal (central) government. Canadian Treasury bills are commonly issued in denominations of $1,000, $5,000, $10,000, $25,000, $50,000, $100,000 and $1 million in Canadian dollars. Canadian Treasury bills are short-term securities that can have maturity from a month to a year. These treasury securities are issued and sold at public auctions. Treasury bills must be purchased, transferred or sold, directly or indirectly, through a participant of the Debt Clearing Service.It should be also pointed out that the (Federal) Government of Canada also periodically issues cash management bills (CMBs). CMBs are Canadian Treasury bills with maturities of less than three months used as a source of short-term financing. The maturity tenor of CMBs can be an be as short as one day.

Core inflation is a measure of inflation that excludes the prices of volatile items, like food and energy, from the price index. The prices of certain CPI components can be rather volatile. Thus core inflation rather than the headline CPI inflation is widely used to gauge the underlying trend of prices. In setting monetary policy, central bankers tend to look through transitory movements in total CPI inflation; instead, they focus on core inflation measures that better reflect the underlying trend of inflation. Two different types of core inflation are measured as the year-over-year percentage changes in: the consumer price index (CPI) excluding food and energy; and the CPI excluding eight volatile components and indirect taxes.

Instead of quarterly GDP, the paper uses the year-over-year percentage change in industrial production in Canada as a measure of economic activity. Industrial production is a measure of output of the industrial sector of the economy, which includes manufacturing, mining, and utilities. The growth of industrial production is a useful gauge of the economic fluctuations and business cycle conditions in an economy.

Two different equity indexes are used, namely the S&P/TSX composite index and the S&P/TSX 60 index. An equity index is the weighted value of the equity prices listed in a stock exchange. It measures the ups and downs of the equity market, based on weighted equity prices of the companies listed in the exchange. The S&P/TSX composite index is the benchmark Canadian equity market index representing roughly 70 percent of the total market capitalization on the Toronto Stock Exchange, whereas the S&P/TSX 60 is designed to measure the large-cap segment of the Canadian equity market.

Two different measures of the exchange rate are also used, namely the spot rates for the CAD per USD (Canadian dollar/US dollar) and the CAD’s nominal effective exchange rate (NEER). The NEER of the CAD measures its value against a weighted average of the currencies of its trading partners. An increase (decrease) in NEER indicates an appreciation (a depreciation) of the Canadian dollar against the weighted basket of currencies of its trading partners.

The central bank’s balance sheet is captured by the total assets of the BOC. The asset side of the BOC consists of its holding of Government of Canada securities, provincial bonds, corporate bonds, securities purchased under resale agreements, derivatives, and all other assets. The central bank’s balance sheet is critical to the functioning of the economy and the financial system as its main liabilities, namely banknotes and commercial bank reserves, are the ultimate means of settlement for transactions. The goals and the policy actions of the central bank affect the size and the growth of its balance sheet.

The natural logarithm is taken for several variables, including the equity index, exchange rate, and the central bank’s balance sheet. The first difference is the natural logarithm of these variables, which is used to obtain the variables’ percentage changes.

A summary of the data is provided in table 1. The first column provides the label for each variable. The second column lists each variable’s description, units, and the date range for the data. The third column displays the data’s frequency, while the final column gives the data’s source(s).

**Table 1:** Data description.

Variable label	Description, data range	Frequency	Source
***Swap yields (SWAP)***			
**CSWAP2Y**	Interest rate swap, 2-year, Canadian dollar, %, January 1998–December 2023	Daily; converted to monthly	Refinitiv
**CSWAP5Y**	Interest rate swap, 5-year, Canadian dollar, %, January 1998–December 2023	Daily; converted to monthly	Refinitiv
**CSWAP10Y**	Interest rate swap, 10-year, Canadian dollar, %, January 1998–December 2023	Daily; converted to monthly	Refinitiv
***Short-term interest rates (STIR)***			
**TBILL3M**	Treasury bill, 3 months, %, January 1998–December 2023	Daily; converted to monthly	Bank of Canada
**TBILL6M**	Treasury bill, 6 months, %, January 1998–December 2023	Daily; converted to monthly	Bank of Canada
***Inflation (INFL)***			
**CPIXFE**	Consumer price index excluding food and energy, %, change, y/y, January 1998–December 2023	Monthly	Bank of Canada, Statistics Canada
**CPIXV**	Consumer price index excluding 8 volatile components and indirect taxes, % change, y/y, January 1998–December 2023	Monthly	Bank of Canada, Statistics Canada
***Economic activity (ACTIVITY)***			
**IPYOY**	Industrial production: manufacturing, mining, and utilities, index, % change, y/y, January 1998–December 2023	Monthly	Statistics Canada
***Equity indexes (EQUITY)***			
**TSX**	S&P/TSX composite index, close price, January 1998–December 2023	Daily; converted to monthly	Toronto Stock Exchange
**TSX60**	S&P/TSX 60 index, close price, January 1999–December 2023	Daily; converted to monthly	Toronto Stock Exchange
***Exchange rates (FX)***			
**USDCAD**	Exchange rate, USD–CAD, average, January 1998–December 2023	Daily; converted to monthly	Bank of Canada
**NEER**	Nominal broad effective exchange rate, Canada, [2010 = 100] January 1998–December 2023	Monthly	JPMorgan
***Balance sheet of the central bank (BS)***			
**ASSETS**	Bank of Canada, balance sheet, total assets, end of period, not seasonally adjusted, January 1998–December 2023	Monthly	Bank of Canada

The summary statistics of all variables in their level and at first difference are provided in the appendix. The appendix also reports the unit root tests of all the variables as per both augmented Dickey-Fuller unit root tests and Kwiatkowski-Phillips-Schmidt-Shin tests. Both types of tests indicate that most of the variables are nonstationary in their levels. All the variables become stationary at their first difference.

## Econometric models and findings

### Econometric models

The paper examines two models under consideration, which are constructed as follows:


$$SWAP_{\text{t}}=\varphi (STIR_{\text{t}},\;INFL_{\text{t}},\;ACTIVITY_{\text{t}})$$ (1)

$$SWAP_{\text{t}}=F(STIR_{\text{t}},\;INFL_{\text{t}},\;ACTIVITY_{\text{t}},\;\Delta LN{\left[EQUITY\right]}_{t},\;\Delta LN{\left[FX\right]}_{t},\;\Delta LN{\left[BS\right]}_{t})$$ (2)

where:

SWAP ∈  {SWAP2Y, SWAP5Y, SWAP10Y},

STIR ∈  {TBILL3M, TBILL6M},

INFL ∈  {CPIXFE, CPIXV},

ACTIVITY ∈  {IPYOY},

EQUITY ∈  {TSX, TSX60},

FX ∈  {USDCAD, NEER},

BS ∈  {ASSETS}, and

LN(.) is the natural logarithm.

Equation (1) models the swap yield based on the short-term interest rate, core inflation, and economic activity, while equation (2) adds the percentage changes in the equity index, exchange rate, and the BOC’s balance sheet to equation (1).

The three different maturity tenors of the long-term swap yields (SWAP) are SWAP2Y, SWAP5Y, and SWAP10Y. The short-term interest rate (STIR) is based on the 3-month and 6-month Treasury bill rates (TBILL3M, TBILL6M). Economic activity (ACTIVITY) is measured by the year-over-year percentage change in industrial production (IPYOY). The equity indices (EQUITY) are from two different indices (i.e., TSX, TSX60). The exchange rate (FX) is based on the bilateral exchange rate (USDCAD) and the nominal effective exchange rate (NEER). The balance sheet (BS) is based on the central bank’s total assets (ASSETS).

### Empirical results

The unit root tests (given in the appendix) indicate that industrial production is not stationary at the level, but all the variables become stationary at their first difference. The Granger causality tests between the Treasury bill rates and swap yields revealed a unidirectional relationship between the various tenors of swap yields and the short-term Treasury bill rate. The results of the Granger causality tests are available upon request.

Following the Granger causality tests, an autoregressive distributed lag (ARDL) is the most appropriate econometric approach for modeling the dynamics of Canadian interest rate swap yields. The ARDL method can be applied whether the regressors are I(1) and/or I(0). In addition, the ARDL method also examines both the short-run and long-run effects of the independent variables on the dependent variable [21]. Moreover, the ARDL approach can incorporate a wide range of lags, as warranted by the dynamics of the data-generating process in the financial markets.

Table 2 and table 3 present the econometric results of the two different models for all three maturity tenors of the swap yield. In the first model, the swap yield is modeled as a function of the short-term interest rate, growth in industrial production, and core inflation. In the second model, the swap yield is modeled with the additional variables of the month-over-month percentage changes in the equity price index, exchange rate, and BOC’s total assets.

**Table 2:** ARDL (p, q) Model (with TBILL3M and CPIXFE).

	CSWAP2Y	CSWAP2Y	CSWAP5Y	CSWAP5Y	CSWAP10Y	CSWAP10Y
	**Main equation**					
**TBILL3M**	0.78*** (0.00)	0.72*** (0.00)	0.60*** (0.00)	0.54*** (0.00)	0.46*** (0.00)	0.42*** (0.00)
**TBILL3M(-1)**	–0.91*** (0.00)	-0.85*** (0.00)	–0.79*** (0.00)	–0.75*** (0.00)	–0.67*** (0.00)	–0.65*** (0.00)
**TBILL3M(-2)**	0.21 (0.13)	0.23 * (0.09)	0.19 * (0.07)	0.27** (0.01)	0.34*** (0.00)	0.38*** (0.00)
**TBILL3M(-3)**	–0.32** (0.05)	-0.32 * (0.07)	–0.13 (0.28)	–0.20 (0.17)	–0.29** (0.01)	–0.26** (0.02)
**TBILL3M(-4)**	0.30*** (0.00)	0.29*** (0.00)	0.16** (0.02)	0.16** (0.03)	0.18** (0.01)	0.14** (0.03)
**CSWAP**_*i*_ **Y(-1)**	1.17*** (0.00)	1.18*** (0.00)	1.22*** (0.00)	1.24*** (0.00)	1.26*** (0.00)	1.26*** (0.00)
**CSWAP**_*i*_ **Y(-2)**	–0.34*** (0.00)	–0.36*** (0.00)	–0.26*** (0.00)	–0.38*** (0.00)	–0.43*** (0.00)	–0.42*** (0.00)
**CSWAP**_*i*_ **Y(-3)**	0.23 * (0.05)	0.24** (0.05)		0.10 (0.12)	0.23** (0.01)	0.14** (0.03)
**CSWAP**_*i*_ **Y(-4)**	–0.14 * (0.07)	–0.13 * (0.08)			–0.08 (0.11)	
**CPIXFE**	0.01 (0.42)	0.01 (0.30)	–0.005 (0.66)	–0.002 (0.86)	–0.01 (0.53)	–0.01 (0.61)
**IPYOY**	0.002 (0.22)	0.003 (0.10)	–0.002 (0.92)	–0.003 (0.90)	–0.002 (0.48)	–0.002 (0.48)
**ΔLNTSX**		0.18 (0.56)		–0.02 (0.94)		–0.21 (0.44)
**ΔLNUSDCAD**		–1.50** (0.02)		–1.85** (0.02)		–1.38** (0.05)
**ΔLNASSETS**		–0.05 (0.67)		–0.17 (0.13)		–0.26** (0.03)
**Intercept**	0.05** (0.05)	0.04 (0.14)	0.06** (0.03)	0.06** (0.06)	0.06 * (0.07)	0.06** (0.06)
	**Cointegrating relationship**					
**Long-term coefficient**	0.88*** (0.00)	0.85*** (0.00)	0.81*** (0.00)	0.76*** (0.00)	0.79*** (0.00)	0.72*** (0.00)
**Rate of adjustment**	–0.08*** (0.00)	–0.07*** (0.00)	–0.04** (0.01)	–0.04** (0.01)	–0.02** (0.04)	–0.03** (0.03)
	**Model information**					
**Obs.**	300	299	300	299	300	299
**Adj R** ^**2**^	0.99	0.99	0.99	0.99	0.99	0.99
**AIC**	–0.89	–0.92	–0.64	–0.66	–0.72	–0.73
	**Diagnostic tests**					
**Joint significance** **F-test**	3023.66 (0.00)	2453.01 (0.00)	2640.41 (0.00)	1891.51 (0.00)	2217.73 (0.00)	1900.16 (0.00)
**Serial correlation** **Durbin-Watson stat**	1.98	2.01	1.95	2.04	1.98	2.01
**Serial correlation Breusch-Godfrey LM test**	0.09 (0.91)	1.07 (0.34)	1.76 (0.17)	1.65 (0.23)	0.11 (0.90)	2.02 (0.13)
**Heteroskedasticity Breusch-Pagan-Godfrey test**	4.22 (0.00)	3.36 (0.00)	3.29 (0.00)	2.23 (0.01)	2.63 (0.00)	2.35 (0.00)
**Normality test** **J-B stat**	46.91 (0.00)	33.63 (0.00)	26.92 (0.00)	17.84 (0.00)	18.93 (0.00)	17.39 (0.00)
**Stability diagnostic** **Ramsey RESET test**	0.22 (0.80)	0.37 (0.69)	0.04 (0.96)	0.01 (0.99)	0.19 (0.83)	0.10 (0.91)

**Note:** *p*-values are in parenthesis. ***, **, and *  implies statistical significance at 1 percent, 5 percent, and 10 percent, respectively. BG LM is with 2 lags and Ramsey RESET test is fitted with 2 terms.

**Table 3:** ARDL (p, q) Model (with TBILL6M and CPIXV).

	CSWAP2Y	CSWAP2Y	CSWAP5Y	CSWAP5Y	CSWAP10Y	CSWAP10Y
	**Main equation**					
**TBILL6M**	1.05*** (0.00)	1.00*** (0.00)	0.88*** (0.00)	0.81*** (0.00)	0.67*** (0.00)	0.63*** (0.00)
**TBILL6M(-1)**	-1.32*** (0.00)	–1.27*** (0.00)	–1.22*** (0.00)	–1.21*** (0.00)	–0.99*** (0.00)	–0.96*** (0.00)
**TBILL6M(-2)**	0.34** (0.02)	0.36** (0.02)	0.30** (0.03)	0.43*** (0.00)	–0.33*** (0.00)	–0.35*** (0.00)
**TBILL6M(-3)**	–0.26 * (0.05)	–0.27 * (0.07)	–0.03 (0.78)			
**TBILL6M(-4)**	0.27*** (0.00)	0.26*** (0.00)	0.10 * (0.09)			
**CSWAP**_*i*_ **Y(-1)**	1.17*** (0.00)	1.18*** (0.00)	1.23*** (0.00)	1.28*** (0.00)	1.25*** (0.00)	1.26*** (0.00)
**CSWAP**_*i*_ **Y(-2)**	–0.34*** (0.00)	–0.37*** (0.00)	–0.27*** (0.00)	–0.40*** (0.00)	–0.35*** (0.00)	–0.37*** (0.00)
**CSWAP**_*i*_ **Y(-3)**	0.24** (0.03)	0.25** (0.03)		0.08 * (0.09)	0.08 (0.13)	0.09 * (0.097)
**CSWAP**_*i*_ **Y(-4)**	-0.16** (0.02)	-0.15** (0.03)				
**CPIXV**	0.005 (0.54)	0.01 (0.42)	–0.01 (0.51)	–0.01 (0.43)	–0.01 (0.40)	–0.01 (0.42)
**IPYOY**	0.001 (0.51)	0.001 (0.39)	–0.001 (0.55)	–0.002 (0.28)	–0.003 (0.15)	–0.003 * (0.10)
**ΔLNTSX60**		–0.06 (0.80)		–0.27 (0.36)		–0.39 (0.14)
**ΔLNNEER**		1.23** (0.03)		1.92** (0.02)		1.36 (0.10)
**ΔLNASSETS**		–0.07 (0.50)		–0.22** (0.05)		–0.27** (0.01)
**Intercept**	0.05** (0.02)	0.04** (0.05)	0.05** (0.06)	0.06** (0.03)	0.05 (0.14)	0.06 * (0.06)
	**Cointegrating relationship**					
**Long-term coefficient**	0.92*** (0.00)	0.91*** (0.00)	0.86*** (0.00)	0.78*** (0.00)	0.84*** (0.00)	0.77*** (0.00)
**Rate of adjustment**	–0.09*** (0.00)	–0.09*** (0.00)	–0.03** (0.01)	–0.03** (0.01)	–0.02 * (0.06)	–0.02** (0.03)
	**Model information**					
**Obs.**	300	299	300	299	300	299
**Adj R** ^**2**^	0.99	0.99	0.99	0.99	0.99	0.99
**AIC**	–1.43	–1.43	–0.92	–0.93	–0.88	–0.88
	**Diagnostic tests**					
**Joint significance** **F-test**	5181.46 (0.00)	4088.61 (0.00)	3506.71 (0.00)	2909.96 (0.00)	3551.17 (0.00)	2602.51 (0.00)
**Serial correlation** **Durbin-Watson stat**	1.96	2.00	1.94	2.06	1.94	1.99
**Serial correlation Breusch-Godfrey LM test**	0.61 (0.54)	1.09 (0.33)	1.73 (0.18)	1.70 (0.19)	1.98 (0.14)	2.98 (0.05)
**Heteroskedasticity Breusch-Pagan-Godfrey test**	4.40 (0.00)	3.96 (0.00)	3.28 (0.00)	3.25 (0.00)	4.20 (0.00)	3.25 (0.00)
**Normality test** **J-B stat**	67.11 (0.00)	67.42 (0.00)	36.08 (0.00)	35.24 (0.00)	19.88 (0.00)	24.63 (0.00)
**Stability diagnostic** **Ramsey RESET test**	0.22 (0.80)	o.58 (0.56)	0.11 (0.90)	0.17 (0.85)	0.26 (0.77)	0.27 (0.76)

**Note:** *p*-values are in parenthesis. ***, **, and *  implies statistical significance at 1 percent, 5 percent, and 10 percent, respectively. BG LM is with 2 lags and Ramsey RESET test is fitted with 2 terms.

Table 2 shows estimations using the 3-month Treasury bill rate, i.e., the main variable of interest. In all models of the three different maturity tenors of swaps, the 3-month Treasury bill rate affects the swap yield positively and significantly. A 100-basis point increase in the 3-month Treasury bill rate increases the 2-year swap yield by 72–78 basis points. This impact is comparable in size to the similar relationship for interest rate swap yields in hard currencies such as the USD, EUR, GBP, and JPY [1,3,4,6,7]. The effect declines with higher maturity terms for the swaps: the impact of the short-term interest rate reduces to 55–60 basis points for the 5-year swaps and to 42–46 basis points for the 10-year swaps. Thus, the longer-term interest rate swap yields denominated in CAD respond less to the BOC’s changes in short-term interest rates.

Table 2 also presents the long-term relationship between the 3-month Treasury bill rate and the swap yield, which does not vary significantly from the 2-year maturity term to the 10-year maturity term, moving from 88 basis points for the 2-year swap to 72 basis points for the 10-year swap. The long-term relationship shows a persistent co-movement of the 3-month Treasury bill rate and swap yields of different maturities. Again, the long-term coefficient between the 3-month Treasury bill rate and swap yields of different maturities follows the patterns found for swaps denominated in other hard currencies, such as the USD [1].

The rate of adjustment to any shock to the long-run relationship between the 3-month Treasury bill rate and the swap yield is generally long and differs significantly for different maturities, dissipating in around 12–33 months. This rate of adjustment is much longer compared to other hard currencies, such as the GBP [3]. This may show that the Canadian swap market is very slow to react to the BOC’s monetary policy decisions.

The 3-month Treasury bill rate has a significant negative lagged effect on swap yields that is larger than the contemporaneous impact, showing a reversion to the trend. In general, the lagged impact of the short-term interest rate shows a longer memory. The swap yields also exhibit a significant impact up to 2–4 lags, showing an autoregressive trend in the models. Among the control variables, only the monthly percentage change in the USDCAD exchange rate showed a negative and significant impact on the swap yields.

A host of post-model information and diagnostic tests are presented in the bottom panel of table 2. The adjusted R^2^ shows a very high degree of explanation for variances in the swap yield by the 3-month Treasury bill rate and its lags, as well as the autoregressive variables. The Akaike information criterion (AIC) also shows a good fit for all models. The joint-significance tests for all models show a rejection of the insignificance of the regressors. The Durbin-Watson statistics and Breusch-Godfrey Lagrange multiplier (LM) tests indicate there is no serial correlation in the error terms in these models. The Breusch-Pagan-Godfrey heteroskedasticity tests reject the null hypothesis of homoscedasticity in all models, showing heteroscedasticity in the errors. However, all the models include the heteroskedasticity- and autocorrelation-consistent standard errors. The Jarque-Bera (J-B) tests indicate the error terms are not normally distributed in all models for all swap term lengths. Lastly, the Ramsey RESET tests indicate that all the models are stable.

Table 3 displays the results of the models of swap yields with some alternative variables. Specifically, these models employ the 6-month short-term Treasury bill rate and a super core inflation (i.e., CPI without eight volatile categories and indirect taxes) instead of the 3-month Treasury bill rate and core inflation, respectively. The control variables also replace the TSX equity index with the TSX60 equity index and employ the nominal effective exchange rate in place of the USDCAD exchange rate. The robustness-checked results in table 3 provide essentially unchanged results. The effects of the 6-month Treasury bill rate on the swap yield are somewhat larger than the 3-month Treasury bill rate. The lagged values of the 6-month Treasury bill rate and the autoregressive terms are very similar to the results in table 2. Among the control variables, the percentage change in the nominal effective exchange rate and percentage change in the BOC’s total assets showed some relationship to the swap yields.

The long-term relationship between the swap yield and the 6-month Treasury bill rate is similar to the results in table 2, albeit a little stronger. The rates of adjustment to any shock to the long-term relationship between the swap yields and 6-month Treasury bill rate is also very similar to those in table 2. The adjusted R^2^, AIC, and post-diagnostic test results are identical to their counterparts in table 2.

## Conclusion

The findings from the estimated models of CAD swap yields have major implications for macroeconomics and finance in Canada. The two different models of swap yields estimated here both show that the current short-term interest rate has a significant effect on swap yields of different maturities. The results hold with alternative independent variables, augmenting the findings. Furthermore, the behavior of CAD swaps is in concordance with the patterns observed in swaps denominated in other currencies, as shown in previous studies cited earlier, lending credence to the plausibility of the Keynesian hypothesis of a strong connection between the short-term interest rate and long-term market interest rates.

These findings also suggest that the BOC has a noteworthy influence on CAD swap yields. These findings vindicate the notion that the BOC has substantial sway over the country’s financial system, as its monetary policy actions influence not just long-term Canadian government bond yields, as [13] had already documented earlier, but also the yields of CAD-denominated interest rate swaps. The results show that relationship between the long-term swap yield and the short-term interest rate is similar to those found for swaps denominated in other hard currencies, such as the USD, GBP, EUR, and JPY [1,3,4,6,7]. The results obtained in this paper can be beneficial to portfolio managers, corporate leaders in multinational business settings, and policymakers, particularly central bankers, for discerning the monetary transmission mechanism and behavior of market interest rates in financial markets.

## Supporting information

S1 Dataset (XLSX)

S1 Appendix Summary statistics. (DOCX)
