# Supplementary material for: Macro-financial models of Canadian dollar interest rate swap yields
Source: PLoS One. 2025 Mar 25;20(3):e0320132. doi: 10.1371/journal.pone.0320132 (PMC11936162; doi:10.1371/journal.pone.0320132)
Supplement: S1 Appendix — (DOCX) [file pone.0320132.s002.docx]

**APPENDIX**

**Summary Statistics**

The summary statistics of all variables in their level and at first difference are presented in table A1 and table A2 respectively. The average swap yields rise with the maturity levels, as longer maturity indicates higher risk. Similarly, the average 3-month Treasury bill rate is lower than the average 6-month Treasury bill rate. The skewness of the swap yields is positive and close to 0.50 or less, showing somewhat symmetrical distributions. The short-term Treasury bill rates have a skewness of just over 0.60, thus showing a slightly fatter tail to the right. The price indices and the Bank of Canada’s (BOC) total assets show significant positive skewness and exhibit a longer tail on the right. The other control variables have smaller negative skewness, revealing a slightly left-sided tail. The kurtosis for the swap yield curve and short-term interest rates is below 3.0, displaying a platykurtic distribution with a short tail (i.e., fewer outliers). A similar short tail can be found for most of the control variables. However, the inflation and core inflation indices, industrial production index, and BOC’s total assets have kurtosis above 3.0 and thus indicate a leptokurtic distribution with a long tail. The Jarque-Bera (J-B) tests in table 2A imply that all the variables examined are not normally distributed, which is common in financial literature.

**Table A1. Summary Statistics of the Variables**

| **Vars.** | **Obs.** | **Mean** | **Std. Dev.** | **Max.** | **Min.** | **Skewness** | **Kurtosis** | **J-B** | **Prob.** |
| --- | --- | --- | --- | --- | --- | --- | --- | --- | --- |
| **CSWAP2Y** | 300 | 2.67 | 1.61 | 6.47 | 0.47 | 0.53 | 2.12 | 23.81 | 0.00 |
| **CSWAP5Y** | 300 | 3.08 | 1.55 | 6.61 | 0.71 | 0.38 | 2.05 | 18.39 | 0.00 |
| **CSWAP10Y** | 300 | 3.55 | 1.50 | 6.76 | 0.96 | 0.24 | 1.92 | 17.52 | 0.00 |
| **TBILL3M** | 300 | 2.06 | 1.64 | 5.68 | 0.06 | 0.63 | 2.09 | 30.23 | 0.00 |
| **TBILL6M** | 300 | 2.16 | 1.66 | 5.93 | 0.10 | 0.61 | 2.08 | 29.18 | 0.00 |
| **CPI** | 300 | 2.21 | 1.38 | 7.87 | -0.95 | 1.50 | 6.61 | 275.84 | 0.00 |
| **CPIXFE** | 300 | 1.88 | 0.97 | 5.48 | 0.52 | 1.91 | 6.75 | 357.27 | 0.00 |
| **IPYOY** | 300 | 1.05 | 4.88 | 19.57 | -21.18 | -0.84 | 5.90 | 140.91 | 0.00 |
| **LNTSX** | 300 | 9.41 | 0.32 | 9.98 | 8.72 | -0.34 | 2.26 | 12.68 | 0.00 |
| **LNTSX60** | 300 | 6.57 | 0.34 | 7.18 | 5.85 | -0.29 | 2.26 | 10.91 | 0.00 |
| **LNUSDCAD** | 300 | 0.22 | 0.14 | 0.47 | -0.05 | -0.15 | 2.02 | 13.17 | 0.00 |
| **LNNEER** | 300 | 4.47 | 0.11 | 4.66 | 4.25 | -0.33 | 2.32 | 11.14 | 0.00 |
| **LNASSETS** | 300 | 11.35 | 0.80 | 13.26 | 10.36 | 1.14 | 3.29 | 65.78 | 0.00 |

Table A2 shows the summary statistics of all the variables at their first difference. The short-term interest rates and swap yields are more volatile at their first difference. The coefficient of variation is much higher than the level data for the variables. The skewness of first-differenced swap yields is negative but less than 0.50, representing a symmetric distribution. However, the first-differenced Treasury bill rates are negative and much larger. Thus, they clearly show longer left-sided tails. All swap yields and short-term interest rates, as well as all the remaining control variables, are leptokurtic. Only the first-differenced core inflation indices exhibit a normal distribution as per the J-B tests.

**Table A2. Summary Statistics of the First Differences of the Variables**

| **Vars.** | **Obs.** | **Mean** | **Std. Dev.** | **Max.** | **Min.** | **Skewness** | **Kurtosis** | **J-B** | **Prob.** |
| --- | --- | --- | --- | --- | --- | --- | --- | --- | --- |
| **ΔCSWAP2Y** | 299 | 0.002 | 0.20 | 0.75 | -0.79 | -0.34 | 4.97 | 54.30 | 0.00 |
| **ΔCSWAP5Y** | 299 | -0.005 | 0.20 | 0.57 | -0.74 | -0.17 | 3.70 | 7.44 | 0.02 |
| **ΔCSWAP10Y** | 299 | -0.006 | 0.19 | 0.54 | -0.80 | -0.15 | 4.01 | 13.88 | 0.00 |
| **ΔTBILL3M** | 299 | 0.001 | 0.18 | 0.55 | -1.12 | -1.81 | 13.22 | 1463.17 | 0.00 |
| **ΔTBILL6M** | 299 | 0.0006 | 0.17 | 0.54 | -1.04 | -1.45 | 10.56 | 816.27 | 0.00 |
| **ΔCPI** | 299 | 0.01 | 0.46 | 1.17 | -1.44 | -0.14 | 3.31 | 2.25 | 0.32 |
| **ΔCPIXFE** | 299 | 0.01 | 0.24 | 0.76 | -0.63 | 0.08 | 3.41 | 2.43 | 0.30 |
| **ΔIPYOY** | 299 | -0.01 | 2.13 | 16.35 | -14.62 | 0.49 | 21.85 | 4438.78 | 0.00 |
| **Δ LNTSX** | 299 | 0.004 | 0.04 | 0.10 | -0.25 | -2.07 | 14.11 | 1750.06 | 0.00 |
| **ΔLNTSX60** | 299 | 0.004 | 0.04 | 0.10 | -0.24 | -1.90 | 12.74 | 1361.00 | 0.00 |
| **ΔLNUSDCAD** | 299 | -0.0004 | 0.02 | 0.11 | -0.06 | 0.53 | 7.36 | 250.78 | 0.00 |
| **ΔLNEER** | 299 | 0.001 | 0.02 | 0.05 | -0.08 | -0.34 | 5.71 | 97.09 | 0.00 |
| **ΔLNASSETS** | 299 | 0.01 | 0.06 | 0.69 | -0.18 | 6.36 | 68.05 | 54742.05 | 0.00 |

**Unit Root Tests**

The unit root and stationarity tests are displayed in table A2 and table A4, respectively. Table A3 exhibits the unit root and stationarity tests of the variables at their level. Results from both the augmented Dickey-Fuller (ADF) unit root tests [1-2] and Kwiatkowski-Phillips-Schmidt-Shin (KPSS) stationarity tests [3] are shown. The null hypotheses for the ADF and KPSS tests are different. The ADF test has a null hypothesis of the presence of a unit root (i.e., non-stationarity), whereas the KPSS test has a null hypothesis of stationarity. The unit root tests indicate most of the variables are nonstationary. The one notable exception is industrial production, which shows the presence of stationarity in both types of tests.

**Table A3. Unit Root and Stationarity Tests of the Variables**

| **Variables at**  **Level** | **ADF Unit Root Tests (H_0_: unit root)** | | | **KPSS Tests (H_0_: stationarity)** | |
| --- | --- | --- | --- | --- | --- |
|  | **None** | **Intercept** | **Trend** | **Intercept** | **Trend** |
| **CSWAP2Y** | -1.18 | -1.90 | -1.38 | 1.12*** | 0.30*** |
| **CSWAP5Y** | -1.22 | -1.87 | -1.82 | 1.54*** | 0.32*** |
| **CSWAP10Y** | -1.16 | -1.71 | -2.03 | 1.66*** | 0.29*** |
| **TBILL3M** | -1.25 | -2.06 | -1.50 | 1.00*** | 0.27*** |
| **TBILL6M** | -1.15 | -1.93 | -1.38 | 1.00*** | 0.27*** |
| **CPI** | -0.38 | -2.21 | -2.34 | 0.28 | 0.19** |
| **CPIXFE** | 0.12 | -1.64 | -1.98 | 0.47** | 0.23*** |
| **IPYOY** | -3.52*** | -3.69*** | -3.68** | 0.09 | 0.08 |
| **LNTSX** | 1.13 | -1.33 | -3.61** | 1.93*** | 0.08 |
| **LNTSX60** | 1.33 | -1.46 | -3.30* | 1.85*** | 0.07 |
| **LNUSDCAD** | -1.02 | -1.65 | -1.49 | 0.53** | 0.45*** |
| **LNNEER** | 0.40 | -1.78 | -1.65 | 0.79*** | 0.44*** |
| **LNASSETS** | 1.33 | -0.69 | -2.46 | 1.71*** | 0.30*** |

**Note:** Significance levels for: *** 1 percent, ** 5 percent, and * 10 percent

Table A4 shows the unit root and stationarity tests of the variables in their first difference. All the variables become stationary at their first difference, as per both ADF and KPSS tests.

**Table A4. Unit Root and Stationarity Tests of the First Differences of the Variables**

| **Variables at**  **First Difference** | **ADF Unit Root Tests (H_0_: unit root)** | | | **KPSS Tests (H_0_: stationarity)** | |
| --- | --- | --- | --- | --- | --- |
|  | **None** | **Intercept** | **Trend** | **Intercept** | **Trend** |
| **ΔCSWAP2Y** | -12.02*** | -12.00*** | -12.09*** | 0.26 | 0.07 |
| **ΔCSWAP5Y** | -12.93*** | -12.92*** | -12.95*** | 0.18 | 0.06 |
| **ΔCSWAP10Y** | -13.49*** | -13.49*** | -13.50*** | 0.14 | 0.06 |
| **ΔTBILL3M** | -6.24*** | -6.23*** | -11.45*** | 0.25 | 0.08 |
| **ΔTBILL6M** | -7.21*** | -7.20*** | -7.34*** | 0.25 | 0.07 |
| **ΔCPI** | -8.36*** | -8.35*** | -8.34*** | 0.03 | 0.03 |
| **ΔCPIXFE** | -7.75*** | -7.77*** | -7.76*** | 0.04 | 0.03 |
| **ΔIPYOY** | -6.62*** | -6.66*** | -6.68*** | 0.02 | 0.02 |
| **Δ LNTSX** | -13.31*** | -13.37*** | -13.34*** | 0.03 | 0.02 |
| **ΔLNTSX60** | -13.09*** | -13.19*** | -13.17*** | 0.03 | 0.03 |
| **ΔLNUSDCAD** | -13.13*** | -13.11*** | -13.13*** | 0.18 | 0.07 |
| **ΔLNEER** | -14.04*** | -14.03*** | -14.03*** | 0.14 | 0.06 |
| **ΔLNASSETS** | -11.12*** | -11.22*** | -11.22*** | 0.07 | 0.04 |

**Note:** Significance levels for: *** 1 percent, ** 5 percent, and * 10 percent

**References**

1. Dickey DA, Fuller WA. Distribution of the Estimators for Autoregressive Time Series with a Unit Root. Journal of the American Statistical Association 1979; 74(366): 427–31. https://doi.org/10.1080/01621459.1979.10482531
2. Dickey DA, Fuller WA. Likelihood Ratio Statistics for Autoregressive Time Series with a Unit Root. Econometrica 1981; 49(4): 1057–72. https://doi.org/10.2307/1912517
3. Kwiatkowski D, Phillips PCB, Schmidt P, Shin Y. Testing the Null Hypothesis of Stationarity Against the Alternative of a Unit Root. Journal of Econometrics 1992; 54 (1–3): 159–78. https://doi.org/10.1016/0304-4076(92)90104-Y
